# Post-Partum Hemorrhage

**Published:** 1874-05

**Authors:** Chas. W. Earle


*THE MEDICAL EXAMINER.—February* 1, 1874.

POST-PARTUM HÆMORRHAGE.—*By Chas. W. Earle, M.D.*—
1. Post-partum hæmorrhage being an exceedingly dangerous conclusion to labor, and liable to take place when we least expect it, we should always be prepared to combat it. We should remain by the bedside of our patient at least one hour after delivery, and should repeatedly satisfy ourselves that the uterus has firmly contracted.

2. Every practitioner should be perfectly familiar with the ordinary methods of treating post-partum hæmorrhage; *he should have ergot with him at confinements*, and, when it is possible, should have ice at hand, in readiness for use.

3. From the known physiological action of electricity, and clinical observation of a few recorded cases, the indications for treatment in uterine inertia are best met, and most safely combated, by the use of the faradic current.

